# CircCNTNAP3-TP53-positive feedback loop suppresses malignant progression of esophageal squamous cell carcinoma

**DOI:** 10.1038/s41419-020-03217-y

**Published:** 2020-11-25

**Authors:** Hui Wang, Xuming Song, Yajing Wang, Xuewen Yin, Yingkuan Liang, Te Zhang, Lin Xu, Feng Jiang, Gaochao Dong

**Affiliations:** 1 The Affiliated Cancer Hospital of Nanjing Medical University, Nanjing, China; 2 Jiangsu Key Laboratory of Molecular and Translational Cancer Research, Cancer Institute of Jiangsu Province, Nanjing, China; 3 The Fourth Clinical College of Nanjing Medical University, Nanjing, China; 4 Department of Thoracic Surgery, Jiangsu Cancer Hospital, Jiangsu Institute of Cancer Research, 210029 Nanjing, China

**Keywords:** Cancer metabolism, Tumour biomarkers, Tumour-suppressor proteins, Non-coding RNAs, Cancer prevention

## Abstract

Mutation or downregulation of p53 (encoded by TP53) accelerates tumorigenesis and malignant progression in esophageal squamous cell carcinoma (ESCC). However, it is still unknown whether circular RNAs (circRNAs), a novel class of endogenous noncoding RNAs, participate in the regulation of this progress. In this study, we explored the expression profiles of circRNAs in three paired samples of ESCC and identified cCNTNAP3, which is a circRNA that originates from the CNTNAP3 gene transcript and is highly expressed in normal human esophageal tissue. However, we found that the cCNTNAP3 expression level was significantly downregulated in ESCC tissues. In vitro and in vivo studies revealed that cCNTNAP3 inhibited proliferation and increased apoptosis in p53 wild-type ESCC cells, but not in mutant cells. Mechanistically, we found that cCNTNAP3 promotes the expression of p53 by sponging miR-513a-5p. Rescue assay confirmed that the suppressive function of cCNTNAP3 was dependent on miR-513a-5p. We also observed that p53/RBM25 participated in the formation of cCNTNAP3, which implied the existence of a positive feedback loop between cCNTNAP3 and p53. Furthermore, the downregulation of cCNTNAP3 was significantly correlated with later T stage and thus can serve as an independent risk factor for the overall survival of patients with p53 wild-type ESCC. In conclusion, the cCNTNAP3-TP53 positive feedback loop may provide a potential target for the management of ESCC, which also reveals the important role of circRNAs in the regulation of p53.

## Introduction

Esophageal carcinoma is one of the most lethal human tumors, and it is the eighth most common cancer and the sixth most common cancer mortality cause worldwide^1^. Nearly half of esophageal carcinoma cases worldwide are diagnosed in China, of which esophageal squamous cell carcinoma (ESCC) constitutes the predominant histology^2^. Although multiple therapies have been used including surgery, chemotherapy, and radiotherapy, the 5-year survival rate of ESCC is still less than 25%^3,4^. Some targeted drugs based on genomics data have been utilized in ESCC, such as gefitinib (an EGFR inhibitor) and programmed death-ligand 1 (PD-L1) blockers^5,6^. However, because few patients benefit from this targeted therapies^7^, it is urgent to explore novel therapeutic targets involved in the progression of ESCC.

It has been reported that more than half of human cancers, including ESCC, have no wild-type p53 function due to deletion or mutation^8^. The important tumor suppressor p53 acts as a transcription factor that plays a very important role in cell processes, including cell-cycle arrest, DNA repair, and apoptosis^9^. In order to maintain the proper function of p53, the level and activity of p53 protein in cells are precisely controlled by a group of positive and negative regulators^10^. Cellular p53 protein levels are under the strict control of MDM2 and MDM4, which are their negative regulators in humans. Recent studies have demonstrated that p53 and its network are also regulated by noncoding RNAs at multiple levels^11^. For example, long noncoding RNA PURPL promotes the progression of colorectal cancer by inhibiting the expression of p53^12^. However, it is still largely unknown whether noncoding RNAs participate in the regulation of p53 expression in ESCC.

As a newly discovered noncoding RNA, circRNAs are a class of covalently closed RNA with a covalently closed loop structure^13^. It has been demonstrated that many circRNAs are aberrantly expressed in various human cancers^14^, including ESCC. Many studies have claimed that circRNAs may play important roles in different diseases and pathophysiological processes, such as mesenchymal stem cell identity maintenance^15^, development^16^, and oncogenesis^17,18^. It has been determined that most circRNAs act as miRNA sponges^19^. Competing endogenous RNA (ceRNA) interaction, also called cross-talk, depends on microRNA response elements (MREs) shared between messenger RNAs and noncoding RNAs^20–22^. For example, circCiRS-7 regulates miR-7 target mRNA expression by acting as an effective miR-7 sponge^23^. However, it has not been reported whether circRNAs maintain tumor progression in ESCC that is dependent on p53 expression.

In our study, we screened circRNA expression in ESCC and identified a novel circRNA, cCNTNAP3, originating from the CNTNAP3 gene transcript. By qRT-PCR, we found that cCNTNAP3 was significantly downregulated in ESCC tissues compared with adjacent normal tissues. Subsequently, functional studies discovered that cCNTNAP3 inhibited proliferation in p53 wild-type ESCC cells. Mechanistically, cCNTNAP3 might serve as a sponge of miR-513a-5p by regulating the expression of wild-type p53. Furthermore, we showed that p53 promotes the biosynthesis of cCNTNAP3 by increasing the expression of RBM25. In this project, cCNTNAP3 and p53 may form a positive feedback pathway to inhibit the malignant progression of ESCC. Our study demonstrates the important role of cCNTNAP3 in the regulation of p53 expression, and it may serve as a novel therapeutic target for the treatment of ESCC patients.

## Materials and methods

### Clinical samples

ESCC tissue and adjacent esophageal tissue were obtained from patients undergoing radical esophagectomy in Jiangsu Cancer Hospital affiliated to Nanjing Medical University. All tissues were immediately frozen in liquid nitrogen and stored at −80 °C. This study was approved by the ethics committee of Nanjing Medical University. Clinical features of the patients are presented in Table S1 and Table S4.

### Microarray analysis

A human circRNA microarray was used for circRNA expression profiling of three pairs of ESCC samples and matched normal tissues. The data has been uploaded to the GEO database. Clinical features of the patients are presented in Table S1.

### Cell culture and treatment

All cell lines (HEEC, Eca-109, TE-1, TE-10, KYSE-450, KYSE-410, and 293T) were purchased from Shanghai Institute of Cell Biology, Chinese Academy of Sciences, and identified by short tandem repeat sequence analysis. All cells were cultured in a humidified environment at 37 °C with 5% CO_2_. TP53 status of these cell lines is presented in Table S2.

### RNA extraction and qRT-PCR

Total RNA of tissue samples and cell lines was extracted by TRIzol reagent (Invitrogen, USA) following the manufacturer’s instructions. Then, the qRT-PCR was performed using SYBR Green Master Mix (Vazyme Biotech). β-actin or U6 was used as internal control when calculated using the ΔΔCt method. All data were analyzed using the StepOnePlus Real-Time PCR System (Applied Biosystems, USA). Primer sequences and their concentrations are shown in Table S3.

### RNase R treatment

Total RNA of Eca-109, KYSE-450, and TE-1 cells was extracted and divided into two groups. One group pretreated RNase R (genie, Guangzhou, China), 3 U/μg RNA, 37 °C, 30 min according to the manufacturer’s instructions. The rest were used as controls, qRT-PCR was then used to detect the expression of cCNTNAP3 and CNTNAP3 treated with or without RNase R. Products were separated on a 2% agarose gel and visualized with GelRed.

### Nuclear and cytoplasmic extraction assay

Nucleocytoplasmic RNA was extracted from the Thermo fisher kit according to the manufacturer’s protocol (Thermo, USA).

### Cell transfection

The siRNAs of target p53 and RBM25 were purchased from Genepharm (Shanghai, China). Lipofectamine iMAX (Thermo, USA) was transfected. Sh-cCNTNAP3 and miR‐513a-5p mimic are provided by RiboBio. The cDNA of cCNTNAP3 and RBM25 were synthesized from Invitrogen and cloned into the expression vector pcDNA3.1. Lipofectamine 3000 (Thermo, USA) was transfected according to the directions. Nucleotide sequences and their concentrations are listed in Table S3.

### Real‐time cell analysis (RTCA)

The RTCA system was applied to monitor cell growth by using cell proliferation plates. After RPMI-1640 or DMEM containing 10% FBS was placed in the chamber, cells (15,000) were plated into each well of the e-plate at 37 °C and 5% CO_2_. Readings were recorded at 15‐minute intervals until the end of the experiment (up to 50 h).

### Clone formation assay

Totally, 1500 cells/wells were seeded on a 6-well plate. Clones are harvested in 10 days. The clones were stained with 1% crystal violet in PBS.

### 5-Ethynyl-2′-deoxyuridine (EdU) assay

Eca-109, KYSE-450, and TE-1 cells were seeded into 96-well templates and harvested at 48 h post transfection. Cells were then incubated with 50 mM EdU for 2 h, fixed with 4% paraformaldehyde, and incubated with Apollo Dye Solution to label proliferating cells. Cell nuclei were counterstained by DAPI. Proliferating cells with green signals were visualized by a Leica DM4000 B LED microscope.

### Animal experiment

All animal experiments were approved by the institutional review committee of Nanjing Medical University (Nanjing, China). They were randomly divided into two groups (*n* = 5, no blinding was performed, respectively). Eca-109 cells transfected with cCNTNAP3 plasmid or control vector (pcDNA3.1) were injected subcutaneously into the axilla of nude mice (1 × 10^7^ cells/mouse). Tumor volume was measured every 2 days after implantation. The mice were killed in 15 days and the tumor was removed and weighed. Some tumor specimens were used to extract protein and total RNA, and the remaining specimens were fixed with 4% paraformaldehyde for 24 h and stained with Ki-67, CD-31, and p53 immunohistochemistry.

### Immunohistochemistry (IHC)

ESCC tissues and xenografts were fixed with 10% formalin and embedded in paraffin. Then, the tissues were cut into 5-μm-thick sections and then incubated overnight with primary antibody anti-p53, anti-Ki-67, and anti-CD-31 (Cell Signaling Technology (CST), USA). The sections were subsequently incubated with an HRP–polymer-conjugated secondary antibody (CST, USA) at 37 °C for 1 h and stained with a 3,3-diaminobenzidine solution. And the p53 status of patients was determined by IHC^24^.

### RNA-binding protein immunoprecipitation (RIP)

A RIP assay was conducted using the Magna RIP Kit (Millipore, USA). A total of 1 × 10^7^ Eca-109 or 293T cells were harvested and lysed and incubated with magnetic beads coated with antibodies against AGO2 or IgG (Millipore, USA) at 4 °C overnight. The beads were washed using a buffer, and the immunoprecipitated RNAs were purified and measured by qRT-PCR.

### Pull-down assay

Biotinylated cCNTNAP3 probes and oligo probes (as negative controls) were designed and synthesized by Genepharm (Shanghai, China) for the pull-down test. Biotinylated cCNTNAP3 probes were incubated with streptavidin magnetic beads (Life Technologies, USA) at room temperature (RT) for 2 h to generate probe coated magnetic beads. The cell lysate was then incubated overnight with microspheres coated with the probe at 4 °C. Then wash the beads. The RNA was extracted with Trizol (Invitrogen) and assessed by qRT-PCR. The sequence of the cCNTNAP3 probe was: 5′-AUAAACCACCUCAGAUUCUCCAAAA-3′. That of the oligo probe was 5′-CACATTGTGCAGATATGCGGGT-3′.

### MiRNA pull down

MiRNA pull-down assay was performed by transfecting Eca-109 and 293T cells with 100 nM 3′-biotinylated miRNA mimics. After 24 h, the cells were washed twice with iced PBS, followed by cell lysis using miRNA Pull down lysis buffer. In the miRNA pull-down assay, biotin-labeled miRNAs were isolated by incubating the beads with 100 μL cell lysate and 100 μL miRNA Pull down lysis buffer at 4 °C for 4 h with rotation. After that, biotin-labeled miRNAs and their interacting RNAs were isolated by Trizol Reagent. Detection of miRNA-interacting RNAs was performed by RT-qPCR.

### Fluorescence in situ hybridization (FISH)

The sections of ESCC tissue or Eca-109 cells (1 × 10^7^) were fixed in 4% paraformaldehyde for 10 min and subsequently washed with PBS. Cells were then permeabilized with 0.5% Triton X-100 in precooled PBS for 15 min at 4 °C. Cy3-labeled cCNTNAP3 probe (5′-ATAAACCACCTCAGATTCTCCCCAAAA-3′) and Fam-labeled miR-513a-5p targeting probe (5′-ATGACACCTCCCTGTGAA-3′) mixture were performed to incubate cells for 4 h at 37 °C. FISH kit (GenePharma China) was used to detect the probe signal according to the instructions. The core is stained with DAPI. These images were taken using a TCS SP5II confocal microscope (Leica Microsystems, Germany).

### Dual-luciferase reporter assay

The p53-binding sites of miR‐RNA were predicted by TargetScan (http://www.targetscan.org). To test the binding specificity, the sequences that interacted with the miR-513a-5p seed sequence were mutated (from GGCCCATATCTGTGAA to CGAATCCCAACCGTT), and the wild-type and mutant p53 3′-UTR was inserted into the pGL3 basic vector (Promega). All vectors were verified by sequencing and luciferase activity was evaluated using the dual luciferase assay kit (Promega) according to the manufacturer’s instructions.

### Western blot analysis

Western blot analyses were carried out according to standard protocols. Anti‐β‐actin, anti-p53, anti-p21, anti-cyclin D1, anti-CDK4, anti-cyclin E1, anti-CDK2, anti-pRb, anti-E2F1, and anti‐caspase3 were purchased from CST.

### Statistical analysis

GraphPad Prism 8.0 Software was used for statistical analysis. Most graphs contain graphs for each data point and show the mean ± standard deviation. To test the significance, a *t* test was carried out, and the *p* value was denoted by an asterisk.

## Results

### Identification and characterization of cCNTNAP3 in ESCC

**Fig. 1:**
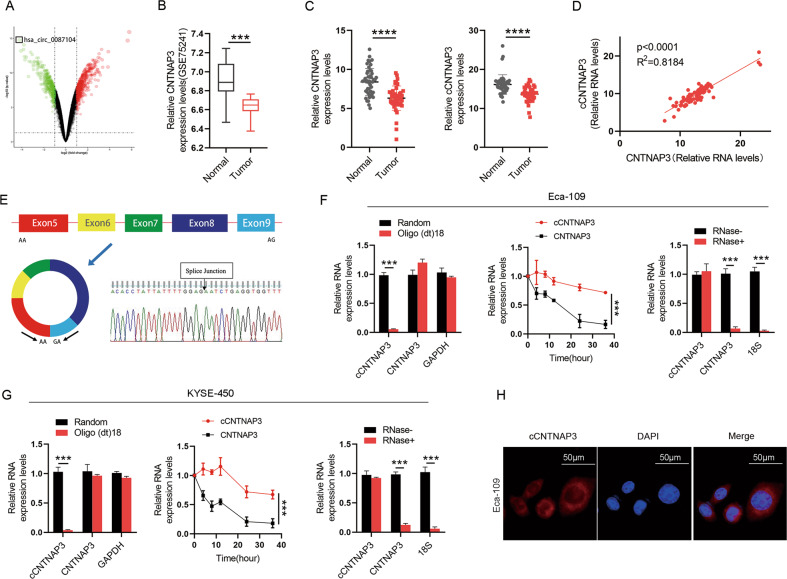
Identification, characterization, and clinical significance of cCNTNAP3 in ESCC. **a** Three pairs of ESCC tissues and matched adjacent normal tissues were analyzed by circRNA microarray and the differentially expressed circRNAs were shown by a volcano plot. **b** CNTNAP3 gene expression in ESCC tissues and matched adjacent normal tissues. (Data from GEO). **c**, **d** CNTNAP3 and cCNTNAP3 expression in 48 pairs of ESCC and adjacent normal tissues was measured by qRT-PCR. **e** cCNTNAP3 originates from back-spliced exons 5 to 9 of CNTNAP3. The back-splice junction of cCNTNAP3 was identified by Sanger sequencing. **f**, **g** cCNTNAP3, and CNTNAP3 mRNA expression levels were measured by qRT-PCR in Eca-109 and KYSE-450 cells. (1) Using random hexamer or oligo (dT)18 primers; (2) after treatment with actinomycin D at the indicated time points; (3) treated with or without RNase. **h** FISH assays showed the location of cCNTNAP3 in Eca-109 cells, scale bars, 50 μm. All the results were shown as mean ± SD (*n* = 3), which were three separate experiments performed in triplicate. *p < 0.05; **p < 0.01; ***p < 0.001; ****p < 0.0001 (Student’s *t* test).

To investigate the differentially expressed circRNAs in ESCC, three pairs of ESCC tissues and matched adjacent normal tissues were analyzed by circRNA microarray (Data has been uploaded to Gene Expression Omnibus (GEO), GSE150476). A total of 223 differentially expressed circRNAs were identified from the analysis, among which 118 were upregulated, and 105 were downregulated. A novel circRNA (cCNTNAP3, CircBase ID: hsa_circ_0087104) derived from exons 5 to 9 of the contactin associated protein-like 3 (CNTNAP3) gene was most significantly downregulated in ESCC (Fig. 1a). Interestingly, we checked its host gene in the U.S. National Center for Biotechnology Information (NCBI) database and found that it was specifically highly expressed in esophageal tissue (Fig. S1A). Then, we verified the expression of CNTNAP3 in the GEO database (GSE75241) and confirmed that it was significantly downregulated in ESCC (Fig. 1b). We suspect that the circRNA transcribed by CNTNAP3 protects esophageal tissue from malignancy and that the loss of cCNTNAP3 may lead to ESCC. Thus, we selected cCNTNAP3 for further study and subsequently measured the expression of cCNTNAP3 and CNTNAP3 in 48 pairs of ESCC and adjacent normal esophageal tissues by qRT-PCR. We found that both CNTNAP3 and cCNTNAP3 expression were significantly lower in ESCC tissues. Moreover, the cCNTNAP3 expression level was significantly correlated with CNTNAP3 mRNA (Fig. 1c, d). The qRT-PCR product of cCNTNAP3 validated the head-to-tail splicing, which later was confirmed by Sanger sequencing (Fig. 1e).

Next, we conducted a series of experiments to verify the characteristics of cCNTNAP3 in Eca-109, KYSE-450, and TE-1 cells. Oligo(dT)18 assay suggested that cCNTNAP3 did not have a poly-A tail. Furthermore, we used actinomycin D to inhibit transcription, and this showed that cCNTNAP3 was more stable than CNTNAP3 mRNA. Subsequently, the RNase R digestion assay revealed that cCNTNAP3 was resistant to RNase R. At last, we found that cCNTNAP3 could be amplified only in cDNA with the use of divergent primers, but not in gDNA (Fig. 1f, g, Fig. S1B–D). We also used FISH and qRT-PCR to determine that cCNTNAP3 was primarily localized in the cytoplasm in ESCC cells (Fig. 1h, Fig. S1E). These results indicated that as a novel circRNA, cCNTNAP3 was stably downregulated in ESCC.

### cCNTNAP3 inhibits tumor progression in vitro and in vivo

**Fig. 2:**
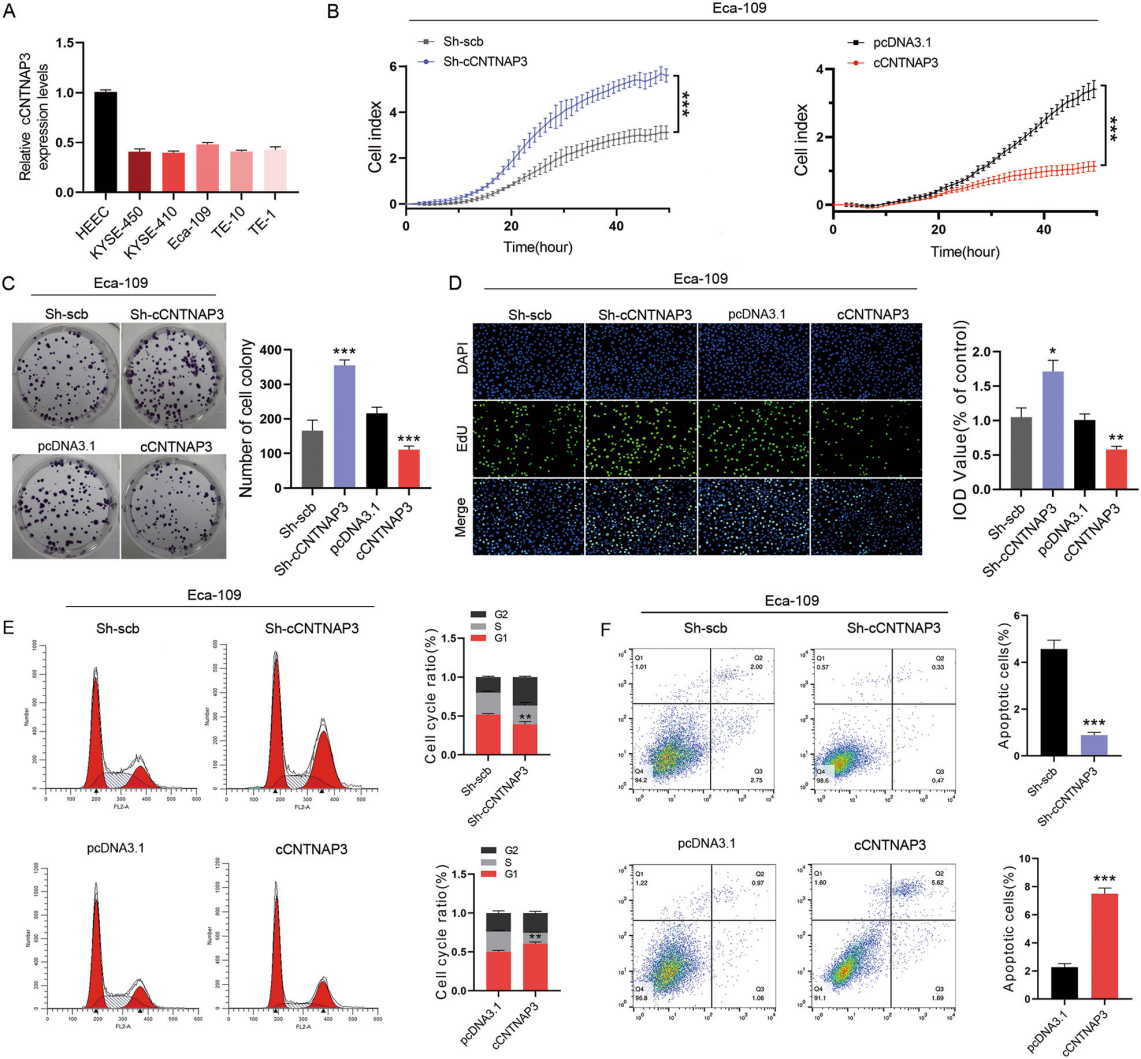
cCNTNAP3 inhibits Eca-109 cell growth and induces cell cycle arrest and apoptosis in vitro. **a** cCNTNAP3 expression in HEEC and multiple ESCC cell lines was measured using qRT-PCR. **b**–**d** RTCA, colony formation, and EdU assays of Eca-109 cells with cCNTNAP3 knockdown or overexpression. **e**, **f** Cell cycle and cell apoptosis rate of Eca-109 cells that received the indicated treatments are analyzed by FACS. All the results were shown as mean ± SD (*n* = 3), which were three separate experiments performed in triplicate. *p < 0.05, **p < 0.01, ***p < 0.001 (Student’s *t* test).

We found that cCNTNAP3 expression levels were lower in all five ESCC cell lines than in HEEC using qRT-PCR (Fig. 2a, Fig. S1F). Then, Eca-109 (p53 wild-type), KYSE-450 (mutant p53 H179R), and TE-1 cells (missense mutation of p53) were selected for cell experiments^25^. We designed shRNAs that could target the back-splice junction of cCNTNAP3, as expected, these shRNAs had no effect on linear CNTNAP3 mRNA levels and sh-cCNTNAP3#3 was selected for further experiments due to its higher inhibitory efficiency (Fig. S2A, B). Furthermore, a cCNTNAP3 overexpressing vector was constructed and transfected into these cells. The expression level of cCNTNAP3 was significantly increased, whereas the linear CNTNAP3 mRNA levels were not affected, as determined by qRT-PCR (Fig. S2C).

RTCA, colony formation, EdU assays showed that knockdown of cCNTNAP3 promoted cell proliferation, while overexpression of cCNTNAP3 suppressed cell growth in Eca-109 cells (Fig. 2b–d). However, we did not observe similar results in KYSE-450 and TE-1 cells (Fig. S2D–F, H–J). The fluorescence-activated cell sorting results also indicated that cCNTNAP3 upregulation increased cell G0/G1 cell arrest and apoptosis, whereas reduced cCNTNAP3 expression led to the opposite results (Fig. 2f, g). As expected, these results were not observed in KYSE-450 or TE-1 cells (Fig. S2G, K). Above all, cCNTNAP3 inhibited proliferation in p53-wild-type ESCC cells, but not in p53-mutant ESCC.

**Fig. 3:**
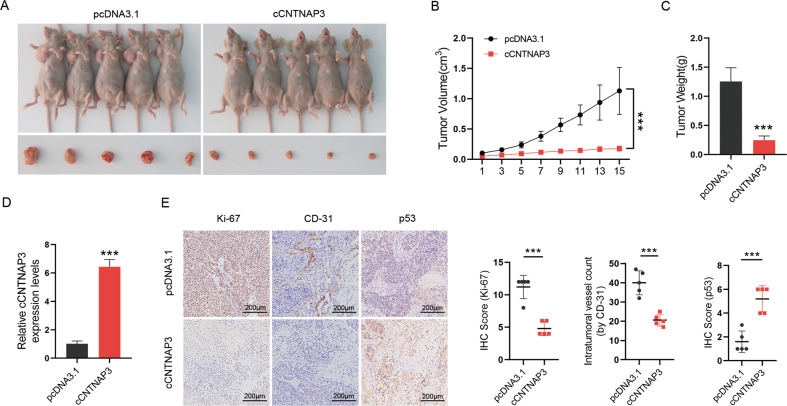
Overexpression of cCNTNAP3 inhibits the growth of ESCC cells in vivo. **a**–**c** Eca-109 cells transfected with empty vector (pcDNA3.1) or cCNTNAP3 plasmids were subcutaneously injected into the armpit of nude mice (1 × 10^7^ cells per mice, *n* = 5 each group). The volume and weight of subcutaneous xenograft tumors of ESCC cells isolated from nude mice. **d** The expression level of cCNTNAP3 in xenografted tumors was analyzed by qRT-PCR. **e** IHC staining showed the protein expression of Ki-67, CD-31, and p53 in xenograft tumor tissues. All the results were shown as mean ± SD (*n* = 3), which were three separate experiments performed in triplicate. *p < 0.05, **p < 0.01, ***p < 0.001 (Student’s *t* test).

To further explore whether cCNTNAP3 is involved in tumorigenesis in vivo, Eca-109 cells, transfected with empty control vector (pcDNA3.1), or cCNTNAP3 were subcutaneously injected into nude mice. The results showed that overexpression of cCNTNAP3 significantly decreased the tumor growth rate and tumor weight compared to the control group (Fig. 3a–c). These subcutaneous tumors were then collected for further study. The qRT-PCR results showed that the expression of cCNTNAP3 in the overexpressed group was significantly higher than that in the control group (Fig. 3d). The IHC demonstrated that Ki-67 and CD-31 were significantly decreased in the cCNTNAP3 overexpression group, while p53 expression was increased after overexpression of cCNTNAP3 (Fig. 3e). Collectively, these results suggested that cCNTNAP3 could suppress the growth of p53 wild-type ESCC in vitro and in vivo.

### cCNTNAP3 acts as a sponge of miR-513a-5p

**Fig. 4:**
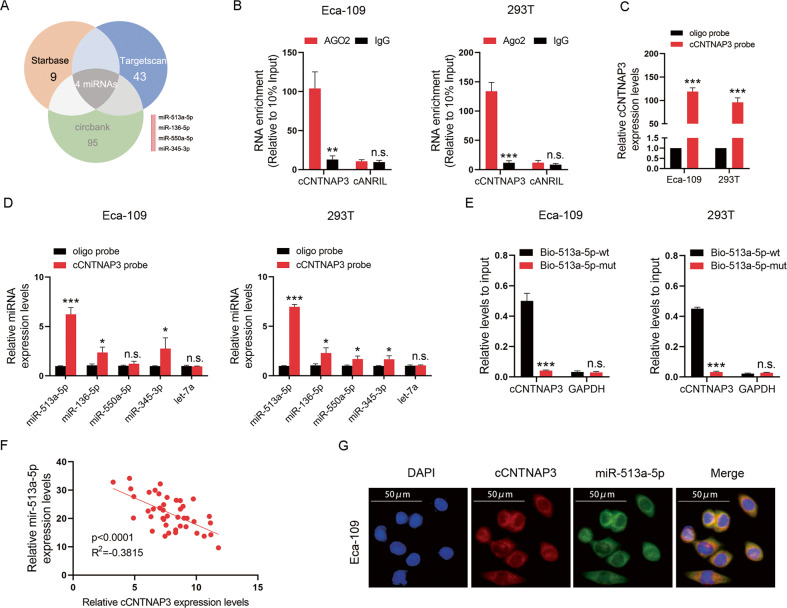
cCNTNAP3 is a sponge of miR-513a-5p. **a** The Venn diagram shows the intersection of miRNA lists. **b** A RIP assay was performed using an AGO2 antibody, and IgG served as a negative control. **c** qRT-PCR results showed that cCNTNAP3 could be specifically enriched by cCNTNAP3 probe. **d** The relative expression levels of four miRNAs candidates were detected by qRT-PCR in Eca-109 and 293T cell lysates. **e** cCNTNAP3 was enriched by biotinylated wild-type or mutant miR-513a-5p, and qRT-PCR was used to determine the relative cCNTNAP3 and GAPDH mRNA levels. **f** Expression of cCNTNAP3 and miR-513a-5p were measured using qRT-PCR in ESCC tissues. **g** RNA FISH images showed the localization of cCNTNAP3 and miR-513a-5p in Eca-109 cells, scale bar, 50 μm. All the results were shown as mean ± SD (*n* = 3), which were three separate experiments performed in triplicate. *p < 0.05, **p < 0.01, ***p < 0.001 (Student’s *t* test).

CircRNAs are known to play a number of important roles, one of which is as a miRNA sponge^18^. We had confirmed that cCNTNAP3 was primarily localized in the cell cytoplasm, to explore the ability of cCNTNAP3 to sponge miRNAs, four miRNAs (miR-513a-5p, miR-136-5p, miR-550a-5p, and miR-345-3p) were screened through bioinformatic analysis databases (Fig. 4a). Next, we used Eca-109 and 293T cells for the RIP experiment. The results showed that cCNTNAP was significantly enriched under the action of anti-AGO2 antibody (Fig. 4b).

To validate whether these candidate miRNAs can directly bind cCNTNAP3, circRNA pull-down assays were performed with a specific biotin-labeled cCNTNAP3 probe. As expect, the biotin-probe can specifically bind cCNTNAP3 (Fig. 4c). Among the four candidate miRNAs, miR-513a-5p was found to be the most abundantly pulled-down by the cCNTNAP3 probe in both Eca-109 and 293T cells (Fig. 4d). We also conducted a biotinylated miR-513a-5p pull-down experiment, the results showed that the enrichment of cCNTNAP3 in the capture portion of mutant miR-513a-5p was significantly reduced compared with that of the wild-type miR-513a-5p (Fig. 4e, Fig. S3A). Furthermore, we found that there is a significant negative correlation (*R*^2^ = −0.3815, *P* < 0.001, *n* = 48) between cCNTNAP3 and miR-513a-5p in ESCC tissues (Fig. 4f). Finally, the FISH analysis showed that miR-513a-5p co-localized with cCNTNAP3 in the cytoplasm (Fig. 4g). In summary, these results demonstrated that cCNTNAP3 could directly bind miR-513a-5p.

### miR-513a-5p reverses the tumor-suppressor role of cCNTNAP3 in p53 wild-type ESCC

**Fig. 5:**
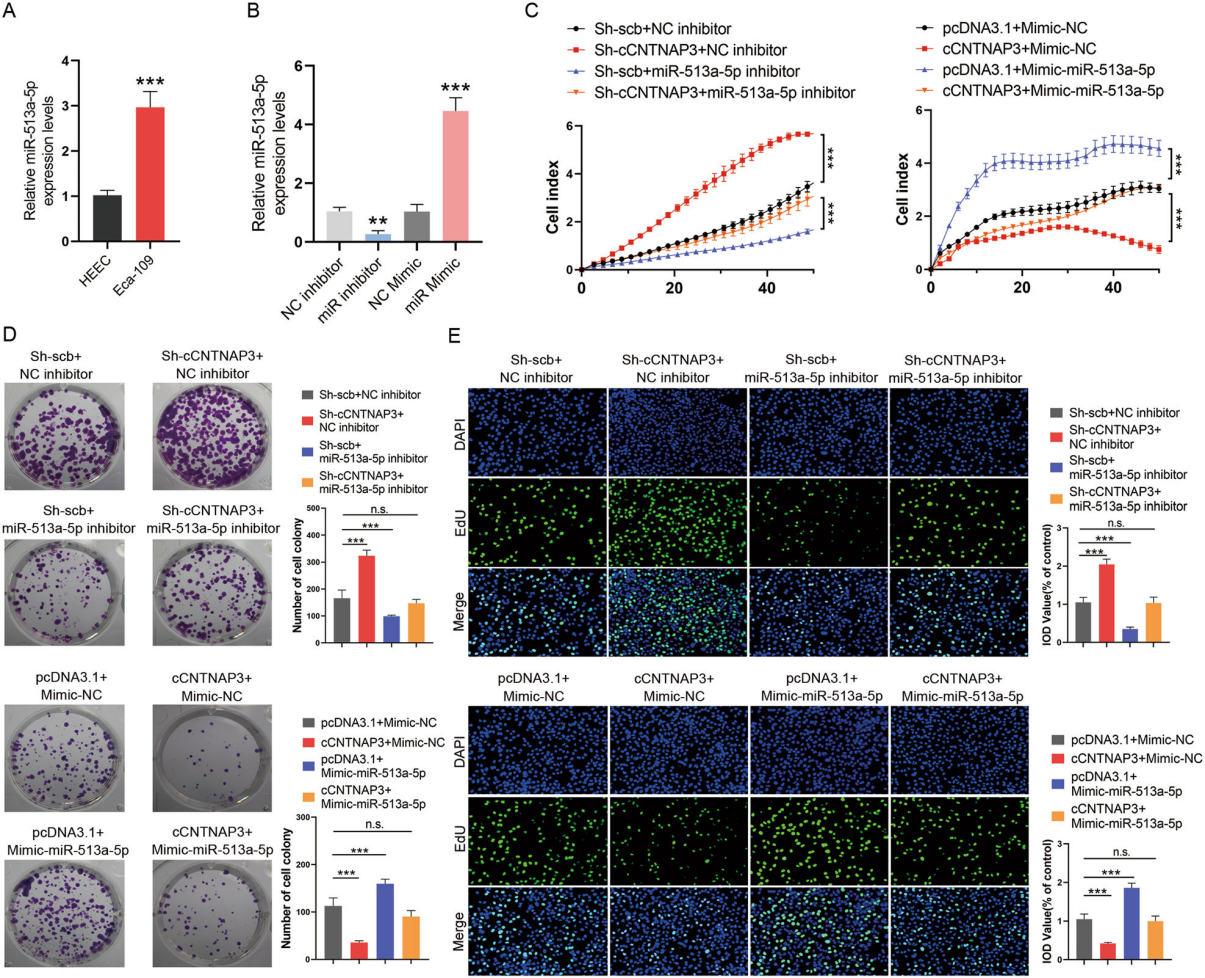
miR-513a-5p reverses the tumor-suppressor roles of cCNTNAP3 in Eca-109 cells. **a** The expression level of miR-513a-5p in Eca-109 cells was analyzed by qRT-PCR. **b** The transfection efficiency of miR-513a-5p inhibitor or mimic was determined by qRT-PCR in Eca-109 cells. **c**–**e** RTCA, colony formation, and EdU assays were performed in Eca-109 cells after cCNTNAP3 knockdown or overexpression and cotransfection with miR-513a-5p inhibitor or mimic. All the results were shown as mean ± SD (*n* = 3), which were three separate experiments performed in triplicate. *p < 0.05, **p < 0.01, ***p < 0.001 (Student’s *t* test).

MiR-513a-5p has been reported to be a risk factor for breast cancer^26^. And we found that the expression of miR-513a-5p was increased in Eca-109 cells (Fig. 5a). To investigate the biological function of miR-513a-5p, the mimic and inhibitor of miR-513a-5p were transfected into Eca-109 cells (Fig. 5b). RTCA, colony formation, and EdU assays revealed that knockdown of cCNTNAP3 successfully promoted cell proliferation, while the promotion of cell proliferation by cCNTNAP3 was reversed by co-transfection with miR-513a-5p inhibitor. In contrast, overexpression of cCNTNAP3 inhibited ESCC cell proliferation, and co-transfection with the miR-513a-5p mimic weakened these effects (Fig. 5c–e). In conclusion, the ability of cCNTNAP3 to inhibit p53 wild-type ESCC progression was reversed by miR-513a-5p.

### cCNTNAP3 sponged miR-513a-5p to regulate p53

Previous studies have found that circRNAs may play important role in tumor progression through the circRNA–miRNA–mRNA signaling pathway^19^. We found that p53 mRNA was a candidate that could be targeted by miR-513a-5p using three publicly available algorithms (Fig. S3B). And the p53 gene is also recognized as one of the important genes associated with cell proliferation.

**Fig. 6:**
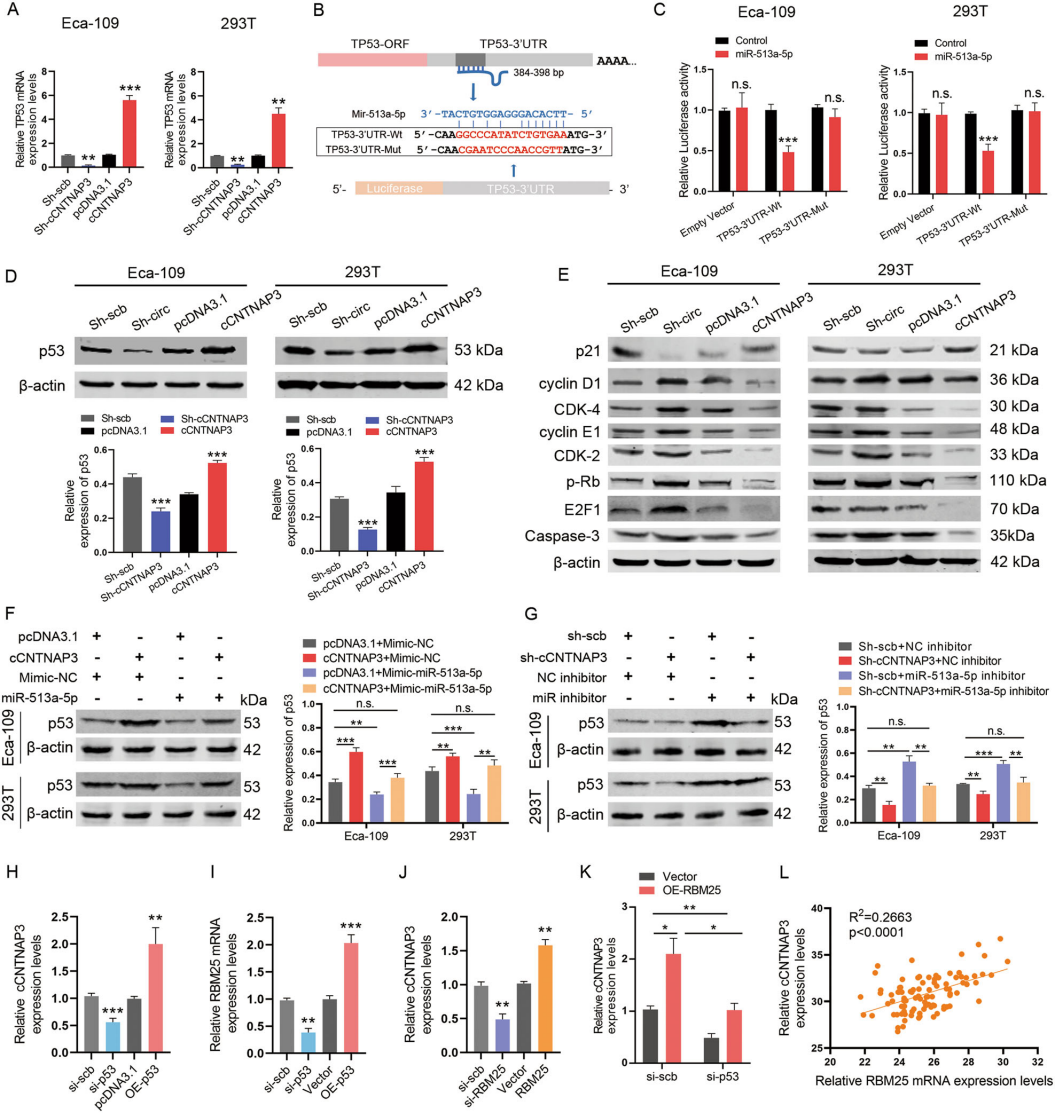
miR-513a-5p promotes the progression of ESCC via targeting p53 and RBM25 mediates p53 regulation of cCNTNAP3 biogenesis. **a** The mRNA level of p53 in Eca-109 and 293T cells after knockdown or overexpression of cCNTNAP3 was determined by qRT-PCR. **b** Upper panel: miR-513a-5p can bind to the 3′-UTR of p53 mRNA; lower panel: Schematic graph illustrated the mutation of potential binding site between miR-513a-5p and the 3′-UTR regions of p53. **c** The direct binding between p53 3′-UTR and miR-513a-5p was analyzed by dual-luciferase reporter assay. **d**, **e** The expression levels of p53 protein and its downstream p21, cyclin D1, CDK-4, cyclin E1, CDK-2, p-Rb, E2F1, and Caspase-3 in Eca-109 and 293T cells with knockdown or overexpression of cCNTNAP3 were detected by western blot. **f**, **g** p53 levels were detected by western blot after cCNTNAP3 knockdown or overexpression and cotransfection with miR-513a-5p inhibitor or mimic. **h** qRT-PCR detected cCNTNAP3 expression level in Eca-109 cells transfected with p53-specific siRNA (si-p53) or p53-overexpression plasmid (OE-p53). **i** Expression level of RBM25 was determined by qRT-PCR in Eca-109 cells after knockdown or overexpression of p53. **j** Expression of cCNTNAP3 was determined by qRT-PCR in Eca-109 cells transfected with RBM25-specific siRNA (si-RBM25) or RBM25-overexpression plasmid (OE-RBM25). **k** qRT-PCR detected the expression of cCNTNAP3 in Eca-109 cells transfected with OE-RBM25 alone or together with si-p53. **l** cCNTNAP3 and RBM25 mRNA levels were measured by qRT-PCR in ESCC tissues. All the results were shown as mean ± SD (*n* = 3), which were three separate experiments performed in triplicate. *p < 0.05, **p < 0.01, ***p < 0.001 (Student’s *t* test).

Next, we used qRT-PCR to measure the p53 mRNA expression levels in Eca-109 and 293T cells that underwent knockdown or overexpression of cCNTNAP3. The results showed that the expression of p53 mRNA was affected by cCNTNAP3 (Fig. 6a). A dual-luciferase reporter assay indicated that co-transfection of miR-513a-5p and p53 wild-type reporter plasmids markedly attenuated the luciferase activity (Fig. 6b, c). These findings confirmed that p53 was indeed a direct target of miR-513a-5p.

After that, we performed western blot assays to determine p53 protein levels after cCNTNAP3 silenced or overexpressed. In Eca-109 and 293T cells, a lower level of p53 was observed in the cCNTNAP3-silenced group, whereas overexpression of cCNTNAP3 led to the opposite results (Fig. 6d). However, the expression level of mutant p53 did not significantly change in KYSE-450 and TE-1 cells (Fig. S4A), it is likely due to the longer banishment period of the mutant p53 protein^27–30^. We also verified that certain proteins were related to the p53 pathway and obtained corresponding results (Fig. 6e).

To further confirm the interaction between cCNTNAP3 and miR-513a-5p, the cCNTNAP3 plasmid and miR-513a-5p mimic were co-transfected into Eca-109 and 293T cells. The level of p53 was significantly increased in the cCNTNAP3+mimic-NC group, but it was reversed when co-transfected with miR-513a-5p mimic (Fig. 6f). Similarly, the expression of p53 was significantly decreased in the sh-cCNTNAP3 group, but this also was reversed when co-transfected with miR-513a-5p inhibitor (Fig. 6g). These results suggest that cCNTNAP3 functions as a miR-513a-5p sponge, regulating p53 expression and inhibiting tumor growth in p53 wild-type ESCC.

### RNA-binding-motif protein 25 (RBM25) mediates the p53 regulation of cCNTNAP3 biogenesis

We then investigated the potential mechanisms for the reduction of cCNTNAP3 in ESCC cells. Interestingly, we used siRNA to knock down the expression of p53 and observed that cCNTNAP3 levels were significantly downregulated in Eca-109 but not in KYSE-450 and TE-1 cells. Reversely, overexpression of p53 significantly increased the expression levels of cCNTNAP3 (Fig. 6h, Fig. S4C).

It has been reported that RBM25 is a direct transcriptional target of p53^31^. A recent report showed that RBM25 regulates a large number of alternating splicing exons throughout the human genome by interacting with the CGGGCA sequence of splicing enhancer exons located within the exon^32^. We also found that cCNTNAP3 contains one binding site of RBM25 (Fig. S4B). Our results also showed that knockdown of p53 inhibited the expression of RBM25 and overexpression of p53 increased RBM25 expression levels (Fig. 6i). Subsequently, we knocked down RBM25 using siRNA (si-RBM25) and used qRT-PCR to examine the expression of cCNTNAP3. The results showed that cCNTNAP3 was significantly downregulated in which RBM25 was knocked down. In addition, RBM25 overexpression significantly increased cCNTNAP3 expression (Fig. 6j).

In further experiments, we overexpressed RBM25 and downregulated p53 expression in Eca-109 cells. We found that overexpression of RBM25 alone increased the expression of cCNTNAP3 compared with the empty vector (pcDNA3.1) transfection, while overexpression of RBM25 and downregulation of p53 eliminated the inductive effect of RBM25 upregulation on cCNTNAP3 expression (Fig. 6k). Furthermore, we found that there was a significant positive correlation (*R*^2^ = 0.2663; *P* < 0.001; *n* = 48) between cCNTNAP3 and RBM25 mRNA in ESCC tissues (Fig. 6l). In summary, these data strongly suggest that RBM25-mediated p53 regulates cCNTNAP3 expression.

### Low cCNTNAP3 expression indicates poor prognosis in p53 wild-type ESCC patients

**Fig. 7:**
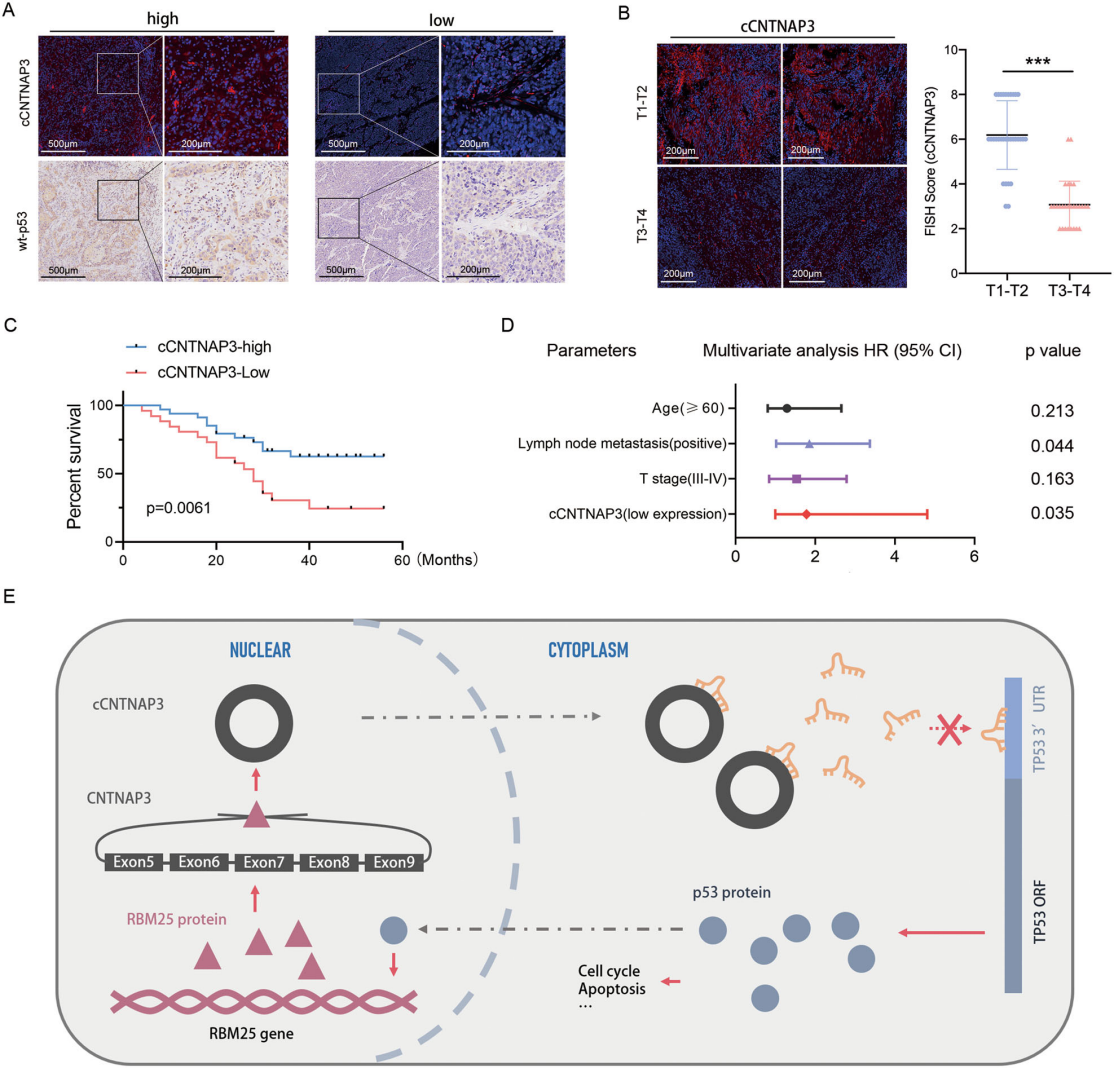
Low cCNTNAP3 expression indicates poor prognosis in p53 wild-type ESCC patients. **a** FISH (cCNTNAP3) and IHC (p53) were performed in a continuous histopathological section of p53 wild-type ESCC tissues. **b** cCNTNAP3 expression was detected in the T1-T4 stage p53 wild-type ESCC tissues using FISH and qRT-PCR. (T1–T2, *n* = 32; T3–T4, *n* = 28). **c** Kaplan–Meier survival analysis of p53-wt patients with higher or lower levels of cCNTNAP3 (*n* = 60). **d** Multivariate analysis of HRs for OS.

We measured the expression of cCNTNAP3 and p53 in 60 p53 wild-type ESCC patients (p53 status determined by IHC) and found there was a positive correlation between cCNTNAP3 expression and p53 protein expression (Fig. 7a). Furthermore, the result also showed a negative correlation between cCNTNAP3 expression and tumor stage. (Fig. 7b). Kaplan–Meier survival curves showed that patients with higher levels of cCNTNAP3 exhibited a longer overall survival (Fig. 7c). Multivariate analyses indicated that low cCNTNAP3 levels are an independent poor prognosis factor for p53 wild-type ESCC patients (Fig. 7d). Our data indicate that low cCNTNAP3 expression indicates poor prognosis in p53 wild-type ESCC patients.

## Discussion

In our study, we found that a novel circRNA (cCNTNAP3), produced by exons 5–9 of the CNTNAP3 gene locus, was significantly downregulated in ESCC compared with paired normal tissues. Furthermore, we searched the NCBI database and found that its host gene is specific to and highly expressed in esophageal tissue. Therefore, we speculated that cCNTNAP3 might be a cancer suppressor gene in the esophagus, and the decreased expression of cCNTNAP3 promotes ESCC development. Subsequent experiments confirmed our idea, where it was observed that overexpression of cCNTNAP3 significantly inhibited p53 wild-type ESCC cell growth and promoted apoptosis, but not in p53 mutant cells. Mechanistically, cCNTNAP3 is able to function as a miRNA sponge to regulate gene expression.

The importance of circRNA in cancer progression has been illustrated in many studies^33,34^. In our study, we found that the downregulation of cCNTNAP3 in ESCC played an important role in promoting the malignant progression of ESCC. The functional role of circRNAs was first described as miRNA sponges^35^. Since then, the function of circRNAs as miRNA sponges has been comprehensively studied in many biological processes. In addition, the importance of circRNA–miRNA interactions has been demonstrated in many cancers, including ESCC^36^. We found that cCNTNAP3 contained the binding sites of several miRNAs, the interaction between cCNTNAP3 and miR-513a-5p in ESCC was further identified by biotinylated RNA pull-down assay, and an RNA FISH assay confirmed that cCNTNAP3 and miR-513a-5p were co-localized in the cytoplasm. Furthermore, a significant inverse correlation occurred between cCNTNAP3 and miR-513a-5p in ESCC tissues. Therefore, it is likely that cCNTNAP3 can serve as a sponge for miR-513a-5p and thus perform a series of functions.

The deletion or mutation of p53 is a crucial feature of ESCC, and it drives tumor progression, metabolism, and metastasis^9,37^. Recent studies have shown that miRNAs interact with p53 and its signaling pathway at multiple levels, and the maladjustment of miRNAs that regulate p53 is considered as an important mechanism leading to the decrease in p53^38^. Subsequently, we confirmed the direct binding of miR-513a-5p and p53 mRNA 3′-UTR by dual-luciferase reporting assay, which indicated that miR-513a-5p regulates the expression of the p53 protein by targeting p53 mRNA. CircRNA is a ceRNA that acts as a miRNA sponge, however, it has not been reported that circRNA adsorbs miRNAs to regulate p53 expression. In our study, we found for the first time that cCNTNAP3 promoted the expression of p53 by sponging miR-513a-5p, which can bind to the 3′-UTR of p53 mRNA, and lead to the degradation of p53 mRNA.

As a transcription factor, p53 regulates the expression of many protein-coding target genes^9^. Recent studies also revealed that p53 can regulate noncoding RNAs expression levels directly or indirectly^39^. The MiR-34 gene family is the first miRNA reported to be directly regulated by p53^38^. In addition, it has been reported that p53 can regulate the expression of circAMOTL1L by promoting the transcription of RBM25^31^. Our study shows that p53 increased the expression of RBM25, and RBM25 promoted the cyclization of cCNTNAP3 and finally increased the expression level of cCNTNAP3.

In this research, we found that cCNTNAP3 inhibited the malignant progression of p53 wild-type ESCC, but it has no obvious function in ESCC with the p53 mutation. Interestingly, we found that cCNTNAP3 reduced the expression level of wild-type p53 protein through sponging miR-513a-5p, but the level of mutant p53 protein did not significantly change after knockdown or overexpression of cCNTNAP3, which is consistent with our previous research results. Furthermore, p53 can promote the generation of cCNTNAP3 through RBM25. At last, we confirmed lower cCNTNAP3 expression indicates a worse prognosis.in p53 wild-type ESCC patients.

In all ESCC patients, the mutation rate of the p53 gene is 55–75%^8,40–42^. Thus, many ESCC patients will still test positive for wild-type p53. For patients without the p53 mutation, the decrease in p53 expression is an important reason for the occurrence and development of ESCC^37^. Our study explains the low expression of p53 in ESCC from one aspect, and also provides a new idea for the treatment of p53 wild-type ESCC. In our experiments, we found that the mutant p53 protein is not significantly regulated by cCNTNAP3, which also reflects the stability of the mutant p53 protein and the complexity of its regulatory mechanism. However, we think cCNTNAP3 is also valuable in ESCC with p53 mutations, and further studies should be performed to obtain more data regarding its mechanisms of action.

## Supplementary information

Supplementary Figure Legends

Supplementary Fig. S1

Supplementary Fig. S2

Supplementary Fig. S3

Supplementary Fig. S4

Supplementary Table S1

Supplementary Table S2

Supplementary Table S3

Supplementary Table S4
