# Supplementary material for: CircCNTNAP3-TP53-positive feedback loop suppresses malignant progression of esophageal squamous cell carcinoma
Source: Cell Death Dis. 2020 Nov 25;11(11):1010. doi: 10.1038/s41419-020-03217-y (PMC7689480; doi:10.1038/s41419-020-03217-y)
Supplement: Supplementary file 1 — Supplementary Figure Legends [file 41419_2020_3217_MOESM1_ESM.docx]

**Fig. S1 a** CNTNAP3 gene expression levels in different human organs was analyzed. (Data from NCBI). **b, c** cCNTNAP3 and CNTNAP3 mRNA expression levels were measured by qRT-PCR in Eca-109, KYSE-450, and TE-1 cells. (1) Use random hexamer or oligo (dT)18 primers; (2) After treatment with actinomycin D at the indicated time points; (3) Treated with or without RNase. **d** As shown by agarose gel electrophoresis, cCNTNAP3 could only be amplified with the divergent primers in cDNA, but not in gDNA. **e** cCNTNAP3 was abundant in the cytoplasm of Eca-109, KYSE-450 and TE-1 cells. GAPDH and U6 were used as positive controls in the cytoplasm and nucleus, respectively. **f** cCNTNAP3 expression levels were measured by qRT-PCR in p53-wt and p53-mut tissue. All the results were shown as mean ± SD (n = 3), which were three separate experiments performed in triplicate. *p<0.05, **p< 0.01, ***p<0.001 (Student’s t test).

**Fig. S2 a** The effects of knockdown or overexpression of cCNTNAP3 in Eca-109 cells were measured using qRT-PCR. **b, c** The expression of cCNTNAP3 and CNTNAP3 mRNA were measured by qRT-PCR in Eca-109, KYSE-450, and TE-1 cells after knockdown or overexpression of cCNTNAP3. **d-f, h-j** RTCA, colony formation and EdU assays of Eca-109 and KYSE-450 cells with cCNTNAP3 knockdown or overexpression. **g, k** Cell cycle of Eca-109 and KYSE-450 cells that received the indicated treatments are analyzed by FACS. All the results were shown as mean ± SD (n = 3), which were three separate experiments performed in triplicate. *p<0.05, **p< 0.01, ***p<0.001 (Student’s t test).

**Fig. S3 a** Schematic graph illustrates the mutation of potential binding site between miR-513a-5p and cCNTNAP3. **b** The Venn diagram shows the genes that miR-513a-5p may bind to.

**Fig. S4 a** Mutation p53 protein expression levels were detected by western blot after konckdown or overexpression of cCNTNAP3. **b** Schematic graph shows the possible binding sites of RBM25 and cCNTNAP3. **c** cCNTNAP3 expression was measured by qRT-PCR in KYSE-450 and TE-1 cells after knockdown of p53. d cCNTNAP3 and p53 mRNA levels were measured by qRT-PCR in p53-wt and p53-mut ESCC tissues. All the results were shown as mean ± SD (n = 3), which were three separate experiments performed in triplicate. *p<0.05, **p< 0.01, ***p<0.001 (Student’s t test).
