# Supplementary figures and images for: CircCNTNAP3-TP53-positive feedback loop suppresses malignant progression of esophageal squamous cell carcinoma
Source: Cell Death Dis. 2020 Nov 25;11(11):1010. doi: 10.1038/s41419-020-03217-y (PMC7689480; doi:10.1038/s41419-020-03217-y)

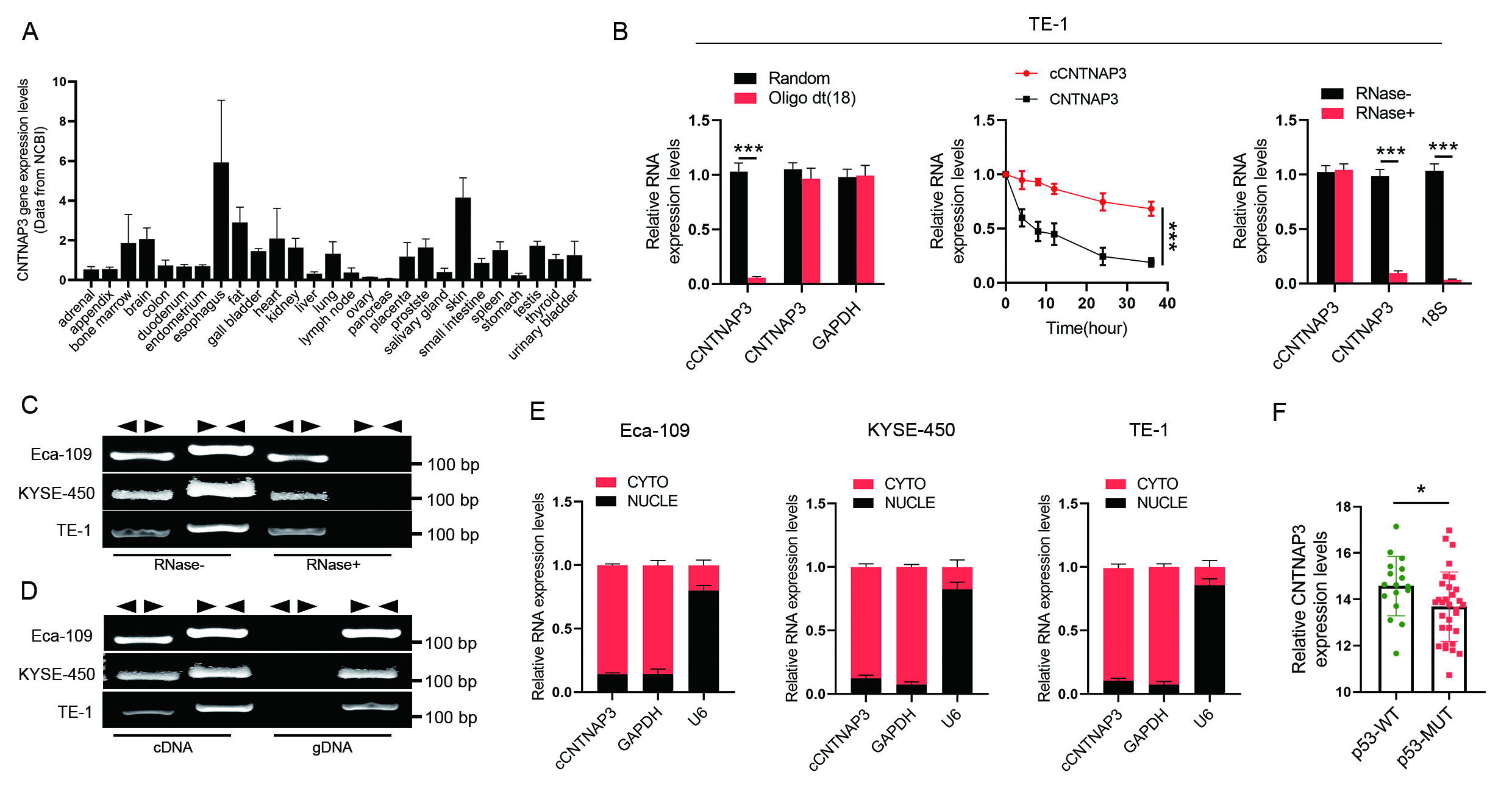

Supplement: Supplementary file 2 — Supplementary Fig. S1 [file 41419_2020_3217_MOESM2_ESM.tif]

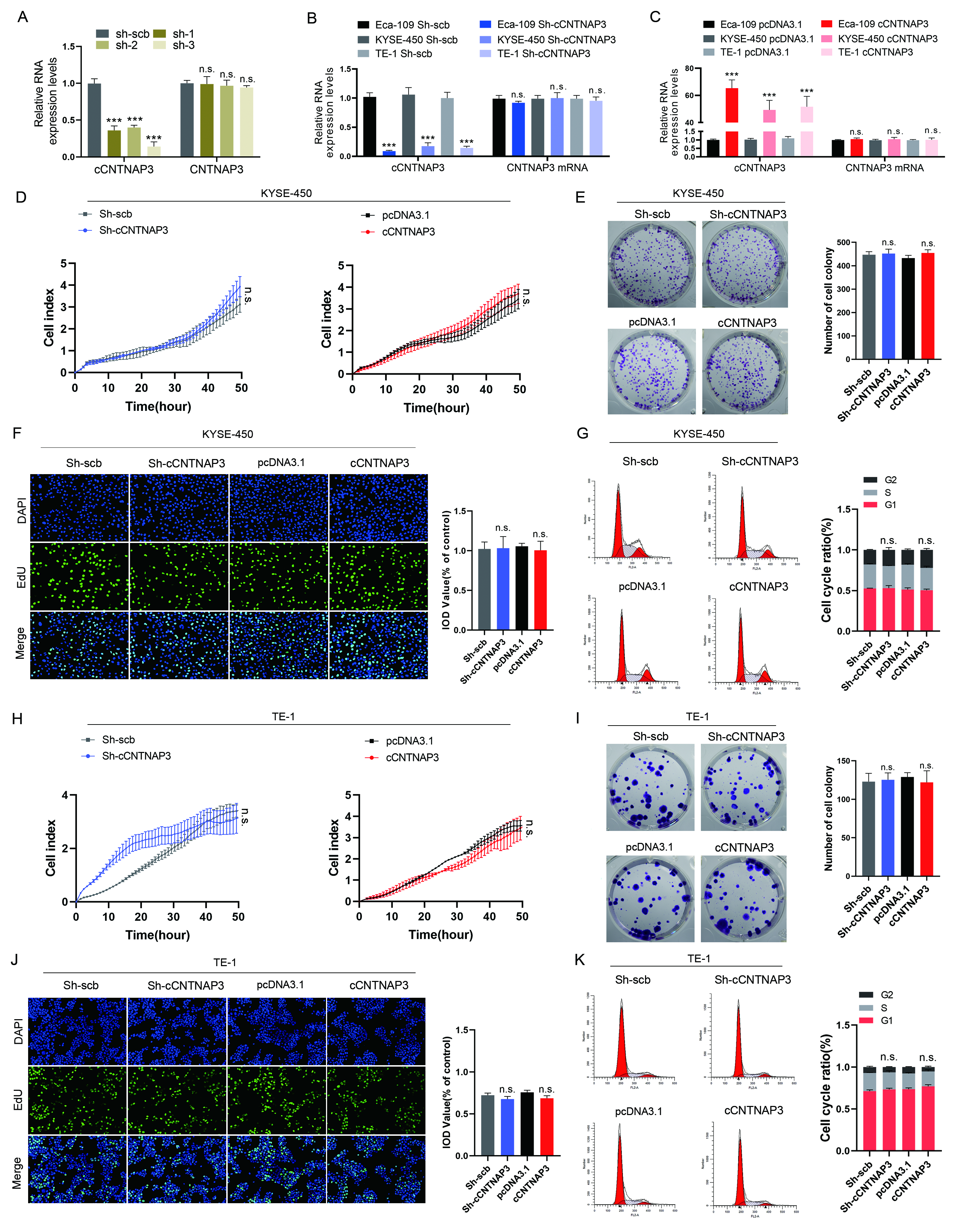

Supplement: Supplementary file 3 — Supplementary Fig. S2 [file 41419_2020_3217_MOESM3_ESM.tif]

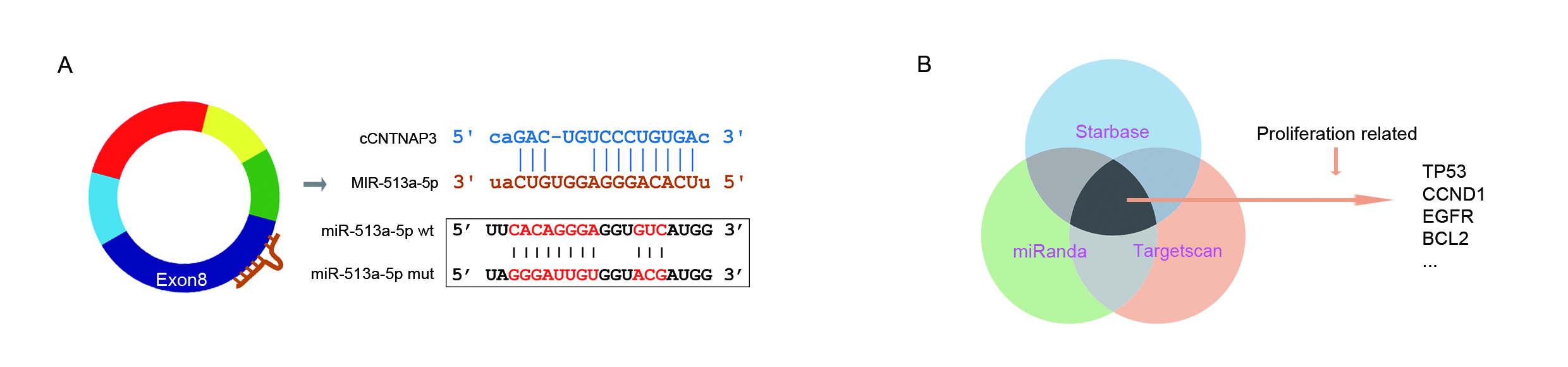

Supplement: Supplementary file 4 — Supplementary Fig. S3 [file 41419_2020_3217_MOESM4_ESM.tif]

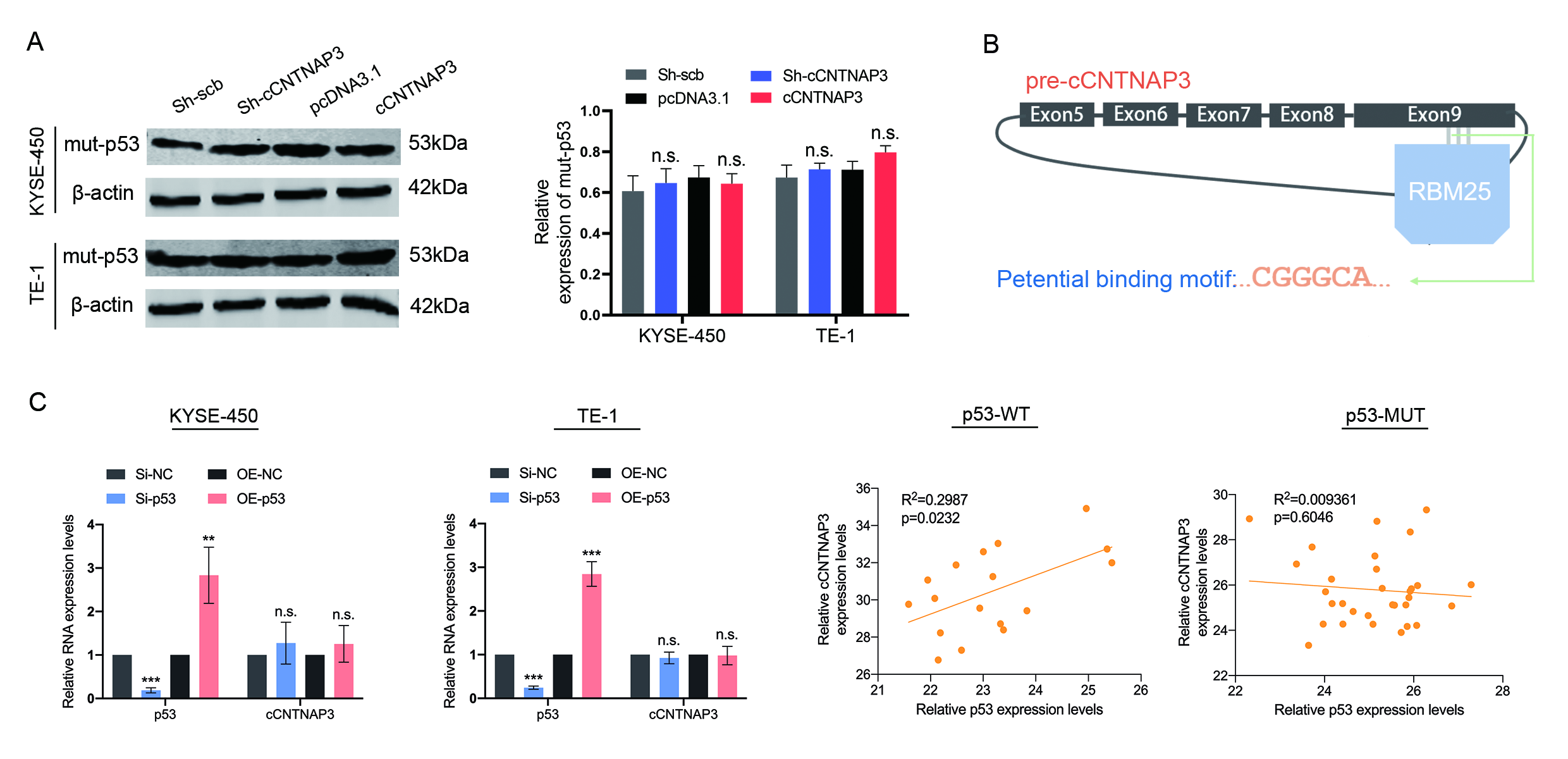

Supplement: Supplementary file 5 — Supplementary Fig. S4 [file 41419_2020_3217_MOESM5_ESM.tif]
