# Supplementary material for: CircCNTNAP3-TP53-positive feedback loop suppresses malignant progression of esophageal squamous cell carcinoma
Source: Cell Death Dis. 2020 Nov 25;11(11):1010. doi: 10.1038/s41419-020-03217-y (PMC7689480; doi:10.1038/s41419-020-03217-y)
Supplement: Supplementary file 7 — Supplementary Table S2 [file 41419_2020_3217_MOESM7_ESM.docx]

| **Cell Lines** | **p53 status** |
| --- | --- |
| HEEC | wild-type |
| Eca-109 | wild-type |
| KYSE-410 | mutant p53 R337Cys |
| KYSE-450 | mutant p53 H179R |
| TE-1 | missense mutation of p53 |
| TE-10 | missense mutation of p53 |
| 293T | wild-type |
