# Supplementary material for: CircCNTNAP3-TP53-positive feedback loop suppresses malignant progression of esophageal squamous cell carcinoma
Source: Cell Death Dis. 2020 Nov 25;11(11):1010. doi: 10.1038/s41419-020-03217-y (PMC7689480; doi:10.1038/s41419-020-03217-y)
Supplement: Supplementary file 8 — Supplementary Table S3 [file 41419_2020_3217_MOESM8_ESM.docx]

| **Table S3: The sequences of primers, oligonucleotides and probes used in this study.** | | |
| --- | --- | --- |
| **Primers for PCR (5’-3’)** | | **concentration** |
| cCNTNAP3-F | ACTCTGTGTCCTTCTCTGCC | 1μmol |
| cCNTNAP3-R | AACCACCTCAGATTCTCCAAAA | 1μmol |
| Liner cCNTNAP3-F | TTCCACGGAGAACTCACTGC | 1μmol |
| Liner cCNTNAP3-R | CCTCACAAGGTCCATTCCCC | 1μmol |
| GAPDH-F | GGAGCGAGATCCCTCCAAAAT | 1μmol |
| GAPDH-R | GGCTGTTGTCATACTTCTCATGG | 1μmol |
| 18S-F | CGAACGTCTGCCCTATCAACTT | 1μmol |
| 18S-R | ACCCGTGGTCACCATGGTA | 1μmol |
| U1-F | ATTGTGGCATTGCGCCGTA | 1μmol |
| U1-R | CGCTCAATCTTTTCCCGTCTTT | 1μmol |
| U6-F | CTCGCTTCGGCAGCACA | 1μmol |
| U6-R | AACGCTTCACGAATTTGCGT | 1μmol |
| cANRIL-F | GCTGGGATTACAGGTGTGAGACACC | 1μmol |
| cANRIL-R | GAATCAGAATGAGGCTTATTCTTCTCATC | 1μmol |
| p53-F | CCAGATGAAGCTCCCAGAATGCC | 1μmol |
| p53-R | CTGTCCCAGAATGCAAGAAGCCC | 1μmol |
| RBM25-F | CGTGCACTCAGATTATTACATGACCTGC | 1μmol |
| RBM25-R | GGGCATTTAGCTCACTGGAGTATTCACG | 1μmol |
| miR-513a-5p-F | Ribobio | 0.2μmol |
| miR-513a-5p-R | Ribobio | 0.2μmol |
| miR-136-5p-F | Ribobio | 0.2μmol |
| miR-136-5p-R | Ribobio | 0.2μmol |
| miR-550a-5p-F | Ribobio | 0.2μmol |
| miR-550a-5p-R | Ribobio | 0.2μmol |
| miR-345-3p-F | Ribobio | 0.2μmol |
| miR-345-3p-R | Ribobio | 0.2μmol |
| **SiRNAs (sense sequence)** | | **concentration** |
| s-cCNTNAP3-1 | AUUAUUUUGGAGAAUCUGATT | 50nmol |
| si-cCNTNAP3-2 | UUUGGAGAAUCUGAGGUGGTT | 50nmol |
| si-cCNTNAP3-3 | GGAGAAUCUGAGGUGGUUUTT | 50nmol |
| si-p53 | GCUCAGAUAGCGAUGGUCUTT | 50nmol |
| si-RBM25-1 | GGAGCUCAGAUCGUAAUAATT | 50nmol |
| si-RBM25-2 | CAGACAUGCUUAUAAGACATT | 50nmol |
| si-RBM25-3 | CUGGAAGAGAGUACAAGGUTT | 50nmol |
| si-scb | UUCUCCGAACGUGUCACGUTT | 50nmol |
|  |  |  |
| **MiR-513a-5p mimics and inhibitors (** **sense sequence)** | | **concentration** |
| mimics NC | UUCUCCGAACGUGUCACGUTT | 50nmol |
| inhibitor NC | CAGUACUUUUGUGUAGUACAA | 50nmol |
| miR-513a-5p mimic | UUCACAGGGAGGUGUCAUGG | 50nmol |
| miR-513a-5p inhibitor | CCAUGACACCUCCCUGUGAA | 50nmol |
| biotin-miR-513a-5p wild-type | UUCACAGGGAGGUGUCAUGG | 50nmol |
| biotin-miR-513a-5p mutant | UAGGGAUUGUGGUACGAUGG | 50nmol |
| **ShRNAs (Vector pGFP-u6)** | | **concentration** |
| sh-cCNTNAP3#1 | CACCGGTGTGCATTCAAGGAAGCCAGCTCGAGCATTATTTTGGAGAATCTGATTTTTTGGATC | 2μg |
| sh-cCNTNAP3#2 | CACCGAAACCGGTTTCAGTGCATTCAAGGAAGCTCGGAGAATCTGAGGTGGTTTTTTGGATC | 2μg |
| sh-cCNTNAP3#3 | CACCGAAACCACCTCAGATTCTCCTTCAAGAGAGGAGAATCTGAGGTGGTTTTTTTTTGGATC | 2μg |
| **Overexpression RNAs (Vetor pcDNA 3.1)** | | **concentration** |
| cCNTNAP3 | hanbio | 2μg |
| p53 | hanbio | 2μg |
| RBM25 | hanbio | 2μg |
